# Bright and stable near-infrared perovskite light emitters supported by multifunctional molecule design strategy

**DOI:** 10.1038/s41377-023-01242-y

**Published:** 2023-09-15

**Authors:** Joo Sung Kim, Tae-Woo Lee

**Affiliations:** 1 SN Display Co. Ltd., Seoul, Republic of Korea; 2 Soft Foundry, Seoul National University, Seoul, Republic of Korea; 3 Department of Materials Science and Engineering, Research Institute of Advanced Materials, School of Chemical and Biological Engineering, Institute of Engineering Research, Seoul National University, Seoul, Republic of Korea

**Keywords:** Organic LEDs, Inorganic LEDs

## Abstract

Perovskite light emitters can realize bright, stable and efficient light-emitting diodes through a molecular design strategy that enables strong endurance on high-current operation.

In a rapidly evolving modern society, the significance of informative and communicative devices, which utilize a diverse range of electromagnetic waves, has seen exponential growth. For instance, visual information, accounting for over 70% of human perception, is deemed one of the most critical applications, thus swift advancement of display devices has been achieved by harnessing visible-range radiation sources (380 ~ 700 nm). Moreover, the exploitation of radiation in the infrared (IR) range (>700 nm) has garnered substantial interest due to its sophisticated applications in night-vision IR imaging, non-destructive bioimaging, optogenetics, optical communication, and laser applications^1–5^. Nevertheless, previous light emitter systems, such as liquid crystals, phosphors, organic emitters, and inorganic quantum dots, have exhibited limitations in their emission characteristics, including luminous efficiency, emission purity, color tunability, and flexibility in material processing and device structure^6^.

Primarily, the emission spectral width, a significant determinant of the purity of the radiometric signal, profoundly influences signal quality. In the context of visible-range display devices, an extremely narrow full-width at half maximum (FWHM) of emitting spectra of ~20 nm is necessitated to express pure color, thereby implementing next-generation ultra-high-definition televisions (UHD-TV). However, this cannot be achieved with the previous light emitter systems of organic light emitters (FWHM ~ 50 nm) or inorganic quantum dots (FWHM ~ 30 nm). Additionally, in the case of bioimaging and optogenetics using the IR-range, as the FWHM broadens and the light intensity weakens, it becomes challenging to provide accurate target energy to the intended energy state. This can result in increased noise levels, low device efficiency, and significant signal loss. This underscores the importance of developing new emitter materials with narrow emission spectra for various optoelectronic devices^6^.

In late 2014, research groups, including those led by Friend et al. and Lee et al., initially reported the potential of room-temperature ultra-narrow emission spectra by employing metal halide perovskite materials as emitting layers in light-emitting diodes (LEDs)^7,8^. The perovskite light-emitting diodes (PeLEDs) exhibit a narrow full-width at half maximum (FWHM, ~20 nm), thus are considered promising candidates for next-generation optoelectronics due to their high emission purity characteristics. However, previous works on PeLEDs have primarily focused on implementing high-efficiency based on a low-dimensional structure, including colloidal nanocrystal or two-dimensional structure with strong carrier confinement, demonstrating high efficiency but low brightness and lifetime^9,10^. Although recent advancements in green-emitting PeLEDs, using non-colloidal in situ core/shell nanocrystals directly synthesized on the substrate, have achieved high brightness (>400,000 cd m^−2^), high efficiency (External quantum efficiency (EQE) > 28%) and long-term stability (>30,000 h at 100 cd m^−2^) simultaneously, the development of IR-emitting PeLEDs is still far from these visible-range PeLEDs^11^. This indicates that device endurance and stability under extensive electric field/voltage are significantly lacking compared to the high brightness and high current density required for the practical use of IR-range devices.

In the issue of Nature on March 15th 2023, Y. Sun and colleagues from the University of Cambridge report the exceptionally high performance of PeLEDs, demonstrating high brightness and long operational lifetime^12^. The resulting PeLEDs provided near-infrared light at 800 nm by utilizing formamidinium lead iodide (FAPbI_3_) as an emitter system, showing a peak EQE of 23.8%, maximum radiance of 663 W sr^−1^ m^−2^, long operational half-lifetime of 32 h at an initial radiance of 107 W sr^−1^ m^−2^ (estimated half-lifetime of >40,000 h at 5 W sr^−1^ m^−2^). The PeLEDs also showed ultra-high radiance over 3200 W sr^−1^ m^−2^ under pulsed operation of ultrahigh current density (4000 mA cm^−2^) with mitigated Joule heating effect, implicating superior endurability under intense electrical stress.

In the study presented here, the authors adopted a multifunctional molecule, 2-(4-(methylsulfonyl) phenyl)ethylamine (MSPE), to achieve an ideal emitter structure with superior opto-electronic, crystal, and morphological properties (Fig. 1). The utilization of the MSPE molecule offered several critical advantages, contributing to the achievement of bright, efficient and stable PeLEDs. Firstly, the homogeneous desired phase with high crystallinity could be achieved by the additive-mediated intermediate slow crystallization process. By suppressing the spontaneous and rapid phase transition into photo-inactive δ-FAPbI_3_, the formation of phase-pure and highly luminescent α-FAPbI_3_ could be achieved, resulting in high EQE in PeLEDs. Secondly, the carrier quenching at the grain boundary and interface of the layer could be suppressed simultaneously. Without any additional process or complex molecule system, the MSPE molecule can combine with each other as well as the surface of the perovskite crystal to form a defect-free layer and interface, thereby achieving an ideal emitting layer without inhomogeneity and non-radiative recombination.

**Fig. 1:**
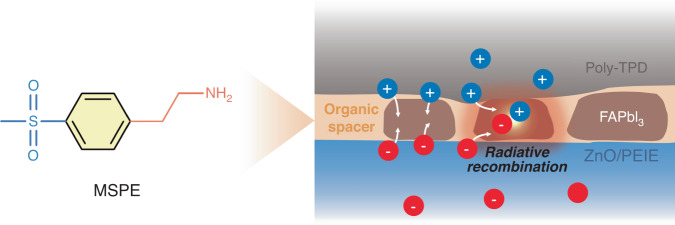
Passivation mechanism in PeLEDs. Molecular structure of the multifunctional molecule, 2-(4-(methylsulfonyl)phenyl)ethylamine (MSPE) (left), and schematic illustration of suppressed non-radiative recombination of perovskite films with MSPE molecules (right)

Furthermore, the authors directly demonstrated the multifunctional role of MSPE molecules by analyzing the effects of phase uniformity on charge-carrier kinetics and spatial inhomogeneity. By using the confocal photoluminescence (PL) spectra imaging method, the authors showed that MSPE molecules can suppress significant spatial variation and obtain homogeneous characteristics in terms of phase, morphology, carrier recombination dynamics, and emission spectra. This advanced analysis technique is the first to directly correlate the morphological and opto-electronic characteristics of PeLEDs, and will be referred to as a major technology that can be essential for various emitter devices in the future.

The realization of PeLEDs with ultra-high brightness, high-efficiency, and long lifetime in this study underscores the feasibility of perovskite emitters in the development of advanced opto-electronic devices. For instance, the high brightness of PeLEDs in this work, exceeding even that of OLEDs or QD LEDs, open possibilities for developing advanced AR/VR displays with high image clarity. This was limited by the low brightness of the display panel compared to that of the external light coming through the viewing window. Also, advanced biomedical applications in deep-tissue imaging or IR-mediated curing could be done with strong IR emission combined with wearable medical devices for health diagnostics, where high power efficiency, flexibility, and strong light intensity are highly desired. As such, the successful development of PeLEDs in this work will pave the way to realize many applications where high brightness and narrow emission spectra are critical, such as high-brightness display panels, AR/VR displays, lasing, and optical networking applications.
